# Crosstalk between *miR-144/451* and Nrf2 during Recovery from Acute Hemolytic Anemia

**DOI:** 10.3390/genes14051011

**Published:** 2023-04-29

**Authors:** Lei Yang, Sheng He, Ling Ling, Fangfang Wang, Lei Xu, Lei Fang, Fan Wu, Shuting Zhou, Fan Yang, Hongwei Wei, Duonan Yu

**Affiliations:** 1 Institute of Translational Medicine, Medical College, Yangzhou University, Yangzhou 225009, China; 2 Jiangsu Key Laboratory of Experimental & Translational Non-Coding RNA Research, Yangzhou University, Yangzhou 225009, China; 3 Guangxi Key Laboratory of Birth Defects Research and Prevention, Guangxi Key Laboratory of Reproductive Health and Birth Defects Prevention, Guangxi Zhuang Autonomous Region Women and Children Care Hospital, Nanning 530000, China; 4 Department of Hematology, Yangzhou University Clinical Medical College, Yangzhou 225001, China; 5 Central Laboratory, Affiliated Hospital of Yangzhou University, Yangzhou University, Yangzhou 225003, China

**Keywords:** *miR-144/451*, *Nrf2*, hemolytic anemia, reactive oxygen species

## Abstract

*miR-144/451* and nuclear factor (erythroid-derived 2)-like 2 (*Nrf2*) regulate two antioxidative systems that have been identified to maintain redox homeostasis in erythroid cells by removing excess reactive oxygen species (ROS). Whether these two genes coordinate to affect ROS scavenging and the anemic phenotype, or which gene is more important for recovery from acute anemia, has not been explored. To address these questions, we crossed *miR-144/451* knockout (KO) and *Nrf2* KO mice and examined the phenotype change in the animals as well as the ROS levels in erythroid cells either at baseline or under stress condition. Several discoveries were made in this study. First, *Nrf2*/*miR-144/451* double-KO mice unexpectedly exhibit similar anemic phenotypes as *miR-144/451* single-KO mice during stable erythropoiesis, although compound mutations of *miR-144/451* and *Nrf2* lead to higher ROS levels in erythrocytes than single gene mutations. Second, *Nrf2*/*miR-144/451* double-mutant mice exhibit more dramatic reticulocytosis than *miR-144/451* or *Nrf2* single-KO mice during days 3 to 7 after inducing acute hemolytic anemia using phenylhydrazine (PHZ), indicating a synergistic effect of *miR-144/451* and Nrf2 on PHZ-induced stress erythropoiesis. However, the coordination does not persist during the whole recovery stage of PHZ-induced anemia; instead, *Nrf2*/*miR-144/451* double-KO mice follow a recovery pattern similar to *miR-144/451* single-KO mice during the remaining period of erythropoiesis. Third, the complete recovery from PHZ-induced acute anemia in *miR-144/451* KO mice takes longer than in *Nrf2* KO mice. Our findings demonstrate that complicated crosstalk between *miR-144/451* and Nrf2 does exist and the crosstalk of these two antioxidant systems is development-stage-dependent. Our findings also demonstrate that *miRNA* deficiency could result in a more profound defect of erythropoiesis than dysfunctional transcription factors.

## 1. Introduction

Mature red blood cells or erythrocytes contain hemoglobin that carries oxygen to tissues where the citric acid cycle is required for energy production. However, oxygen itself and heme moieties in hemoglobin put erythrocytes in danger through oxidative damage [1,2], which could cause severe hemolytic anemia if the tight regulation of antioxidant response is disrupted [3]. *miR-144/451*-mediated and nuclear factor (erythroid-derived 2)-like 2 (Nrf2)-controlled programs are two systems that maintain redox homeostasis in oxygen- and heme-rich erythroid cells by removing excess reactive oxygen species (ROS) from cells [4,5,6,7]. 

*miR-144/451* is a bicistronic gene locus encoding two highly conserved mature *miRNAs*: *miR-451*a and *miR-144*-3p (hereafter referred to as *miR-451* and *miR-144*, respectively) [8]. *miR-451* and *miR-144* abundantly and almost exclusively exist in erythroid cells [8,9,10]. We have previously found that *miR-144/451* gene knockdown in zebrafish and knockout (KO) in mice leads to severe anemia upon acute oxidative hemolysis of red blood cells induced by three strong oxidants: 1-phenyl-2-thiourea (PTU), hydrogen peroxide (H_2_O_2_) or phenylhydrazine (PHZ) [4,11]. Loss of *miR-144/451* directly derepresses 14-3-3z expression, frees 14-3-3z-binding partner Foxo3 and allows Foxo3 to translocate from the cytoplasm into the nucleus [4]. This process activates the transcription of anti-oxidative enzyme catalase (*Cat*) and glutathione peroxidase 1 (*GPX1*) [4]. These findings indicate that *miR-144/451* is an upstream gene required for sustaining Foxo3-mediated amelioration of oxidative damage during erythropoiesis.

Nrf2 is a master regulator of antioxidative responses by inducing the expression of a variety of genes responsible for detoxification and antioxidant activities [12,13]. Targeted disruption of *Nrf2* expression in mice causes hemolytic anemia in an age-dependent manner and *Nrf2* KO erythrocytes are highly sensitive to H_2_O_2_-induced hemolysis both in vitro and in vivo [5,6,14]. These findings indicate the pivotal role of Nrf2 in protecting erythroid cells in pro-oxidant stress environments. 

Though both *miR-144/451* and Nrf2 control antioxidant systems, and mice deficient in either *miR-144/451* or *Nrf2* developed acute anemia when exposed to oxidative stress, three questions remain unanswered: whether a crosstalk occurs between these two systems during erythropoiesis; what the consequences of the interplay between these two systems are; and which system contributes more to protecting animals against hemolytic anemia. To address these issues, we crossed *miR-144/451* KO mice with *Nrf2* KO mice and surprisingly found that although the combined disruption of these two antioxidant systems leads to higher ROS levels in erythrocytes, a compound effect on the erythropoiesis does not appear until double-mutant mice are exposed to PHZ. Interestingly, non-coding *miR-144/451* has a more profound effect on PHZ-induced hemolytic anemia than the Nrf2 transcription factor.

## 2. Materials and Methods

### 2.1. Animals

*miR-144/451* KO (mKO) mice lacking a 388-base-pair (bp) DNA sequence containing the *miR-144/451* locus was described previously [4]. *Nrf2* KO (NKO) mice were generated originally by Dr. Masayuki Yamamoto’s group (Tohoku University Graduate School of Medicine) and gifted by Dr. Depei Liu from the Peking Union Medical College [1]. *Nrf2* KO mice were crossed with *miR-144/451* KO mice to generate double-heterozygote mice and the progenies of double-heterozygote mice were used to generate double-homozygote KO (*Nrf2*^-/-^/*miR-144/451*^-/-^, N/mKO) mice. For genotyping *miR-144/451* KO mice, genomic DNA isolated from mouse tails was amplified by polymerase chain reaction (PCR) with a mixture of 3 primers: Forward (F), 5′- TTC TGC CTG TAA CTC TGG ATC CCT AAG AGA-3′; Reverse 1 (R1), 5′- GGG TAC CCA GAC TAG TAC ATC ATC TAT A-3′; and Reverse 2 (R2), 5′- ATC CCC TCG AGG GAC CTA ATA ACT TC′. For genotyping *Nrf2* KO mice, the following primers were used for PCR with tail DNA as the template: F, 5′- TGG ACG GGA CTA TTG AAG GCT G -3′; R1, 5′- GCC GCC TTT TCA GTA GAT GGA GG -3′; and R2, 5′-GCG GAT TGA CCG TAA TGG GAT AGG-3′. All mice we used in our experiments for this manuscript were 9-10-week-old males. All animal experiments complied with the protocols approved by the Animal Care and Use Committees of the Yangzhou University in China. 

### 2.2. Hematologic Analysis

For erythrocyte indices, blood from adult mice was sampled into tubes coated with EDTA and analyzed on a Hemavet HV950FS analyzer (Drew Scientific, Dallas, TX, USA). Mice were treated with PHZ (Aladdin, Shanghai, China) at a single dose of 50 mg/kg through intraperitoneal (i.p.) injection. Animal survival was closely monitored. Blood smear from peripheral blood was stained with Wright-Giemsa (BA-4017, Baso, Zhuhai, China) according to the manufacturer’s recommendations.

### 2.3. Histology 

Spleens isolated from adult mice were fixed in 4% paraformaldehyde and embedded in paraffin. Hematoxylin and eosin (H&E) staining was performed on four-micron-thick tissue sections. Images were acquired with a microscope (Nikon, 80I, Tokyo, Japan) with 4×, 20× and 100× objective lens, and 10× eyepieces lens.

### 2.4. Flow Cytometry

Cells from peripheral blood, bone marrow and spleen were collected and suspended in phosphate-buffered saline (PBS). Samples were subsequently stained with anti-mouse Ter119 (0.2 μg/10^6^ cells) and anti–mouse CD71 (0.1 μg/10^6^ cells) at room temperature for 20 min. Cells were washed with PBS and analyzed within 1 h using FACSVerse or LSRFortessa Cell Analyzer System (BD Biosciences, Franklin Lakes, NJ, USA). APC-labeled rat anti-mouse Ter119 (557909) and PE-labeled rat anti-mouse CD71 (553267) were purchased from BD Biosciences.

### 2.5. ROS Detection

Blood cells (1 × 10^7^) from mouse tail vein were incubated with 10 μM 2′,7′-Dichlorofluorescin (DCFH-DA, Invitrogen, Carlsbad, CA, USA) for 25 min at 37 °C and analyzed using flow cytometer. Unstained cells were used as negative controls.

### 2.6. Reticulocyte Count

An amount of 2 μL peripheral blood was incubated with 500 μL Retic Count Reagent (Cat#349204, BD Biosciences) at room temperature for 30 min. Samples were analyzed using flow cytometer.

### 2.7. Purification of Erythroblasts

Splenic cell suspension was incubated with 2 mL red blood cell (RBC) lysis buffer (R1010, Solarbio, Beijing, China) on ice for 5 min to remove mature RBCs. Isolation of erythroblasts was performed according to the manufacturer’s recommendations (130-049-901, Miltenyi Biotec, Cologne, Germany). Briefly, cells were incubated with 10 µL anti-Ter119 MicroBeads (130-049-901, Miltenyi Biotec, Cologne, Germany) per 10^7^ cells in 100 μL PBS containing 2% fetal bovine serum (FBS) for 20 min at 4 °C. Cells were resuspended in 500 µL PBS after washing, and loaded into the LS separation column (130-042-401, Miltenyi Biotec). The column was removed from the separator after washing with PBS and the Ter119-labeled cells were flushed out from the column. The Ter119^+^ cell fraction was collected for RNA extraction or preparation for protein lysate.

### 2.8. Quantitative Real-Time Polymerase Chain Reaction (qRT-PCR)

Total RNA was extracted with TRIzol reagent (Invitrogen). An amount of 1 μg of RNA was used for reverse transcription using the Primer Script RT reagent Kit (Takara, Japan). qRT-PCR was performed using the TB Green Premix ExII Taq II (Takara, Japan) and conducted on a Roche or 7500 Real Time PCR system (Applied Biosystems, White Plains, NY, USA). Relative mRNA levels were calculated based on ΔΔCt method. *Gapdh* was used as internal control. All reactions were conducted in triplicate. Primers for qRT-PCR and the sequences are listed in Supplementary Table S1.

### 2.9. Western Blot

The erythroblasts sorted from spleen were lysed with cell lysis buffer (KGP701, KeyGEN Biotech) containing freshly prepared protease inhibitor for 30 min on ice. The supernatant was collected after centrifugation for 15 min at 4 °C. The loading buffer was added to the supernatant and samples were denatured on a metal heater at 95 °C for 5 min. The protein was separated with 12% SDS-PAGE and transferred onto PVDF membrane (1620177, Bio-Rad, Hercules, CA, USA) with 100 V for 2 h. After blocking with 5% skim milk in Tris-buffered saline Tween (TBST) solution for 1.5 h at room temperature, the membrane was incubated with 14-3-3z (1:600, sc-1019, Santa Cruz Biotechnology, Santa Cruz, CA, USA) and GAPDH (1:5000, ab181602, Abcam, Cambridge, UK) antibodies overnight at 4 °C. The membrane was washed with TBST 3 times and incubated with a secondary antibody (1:5000, ab205718, Abcam) for 1 h at room temperature. ECL reagent (WBKlS0100, Millipore, Boston, MA, USA) was used to show protein bands and ImageJ software was used for quantitative analysis of the intensity.

### 2.10. Statistical Analysis 

Microsoft Excel or Graphpad Prism 7 software were used to produce graphs and carry out statistical analyses. Data from 3 experiments or at least 3 different samples were presented as the mean ± standard deviation. Statistical differences were assessed by a two-tailed Student’s *t* test. *p* < 0.05 is considered statistically different, and *p* < 0.01 indicates significantly different.

## 3. Results

### 3.1. Nrf2/miR-144/451 Double-KO Mice Display Similar Hemolysis Levels to miR-144/451 Single-KO Mice though Double-KO Mice Generate More ROS at Baseline

**Figure 1:**
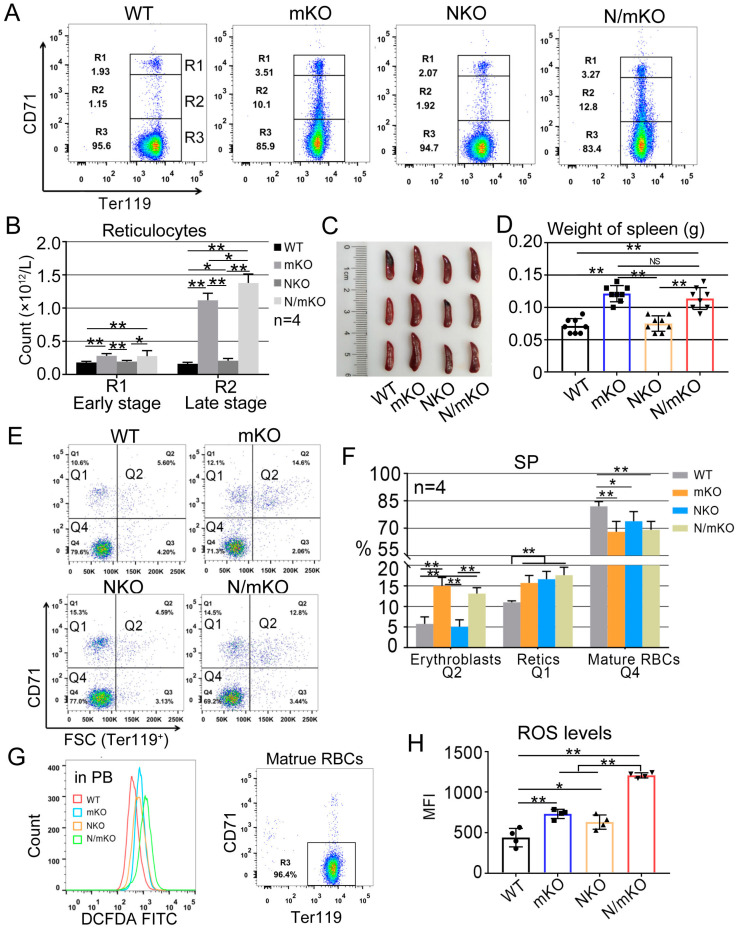
Mouse phenotype change at baseline. (**A**) Flow cytometric analysis of Ter119 and CD71 double-stained cells in peripheral blood of wild-type (WT), *miR-144/451* knockout (mKO), *Nrf2* knockout (NKO) and *Nrf2/miR-144/451* double-knockout (N/mKO) mice. (**B**) Quantitative analysis of early stage (R1) and late stage (R2) of reticulocytes in four types of mice. Note: reticulocyte count = reticulocyte percentage × total RBC number. * *p* < 0.05; ** *p* < 0.01 (*t*-test). (**C**) Spleens from 10-week-old mice. (**D**) Quantitative analysis of spleen weight from 8 mice of each group. ** *p* < 0.01 (*t*-test). (**E**) Flow cytometry showing the cell size of Ter119-positive cell populations in spleens (SP) based on forward scatter channel (FSC). CD71^+^/FSC^high^ (Q2 region) cells represent erythroblasts; CD71^+^/FSC^low^ (Q1 region) cells represent reticulocytes (Retics); CD71^−^/FSC^low^ (Q4 region) cells are largely mature erythrocytes. (**F**) Percentages of cells in different regions based on flow cytometric analysis from panel E. * *p* < 0.05; ** *p* < 0.01 (*t*-test). (**G**) ROS levels in gated Ter119^+^/CD71^−^ mature red blood cells which were measured using the fluorescent indicator 2′,7′-Dichlorofluorescin diacetate (DCFDA). (**H**) Quantitative analysis of the mean fluorescence intensity in all the samples from panel G. Note: *Nrf2* deficiency synergizes *miR-144/451* gene deletion in ROS accumulation in peripheral red blood cells. n = 4 per group. * *p* < 0.05; ** *p* < 0.01 (*t*-test).

Since antioxidation in erythroid cells is orchestrated by systems including Nrf2 and *miR-144/451*, we evaluated the effect of the combined loss-of-function mutation of these two genes by crossing *Nrf2* KO and *miR-144/451* KO mice. We first confirmed that during stable erythropoiesis, *miR-144/451* KO mice, compared to wild-type (WT) animals, displayed a mild microcytic anemia characterized by a small but significant reduction in RBC count, hemoglobin level and hematocrit (HCT), and an increase in RBC distribution width (RDW), reticulocyte count and spleen weight (Table 1, Figure 1A–D). Flow cytometric analyses further demonstrated higher percentages of the CD71^+^/FSC^high^ fraction of Ter119^+^ erythroid cells (nucleated erythroid cells) in *miR-144/451* KO spleens (Figure 1E,F), suggesting a differentiation defect of erythroblasts in *miR-144/451* KO animals. These results support our previous finding that knocking out the *miR-144/451* gene leads to anemia partially due to a differentiation block [15]. Surprisingly, no apparent phenotype changes were found in *Nrf2* KO mice based on the complete blood count (CBC), flow cytometric analyses and spleen size (Table 1, Figure 1A–F). 

To investigate whether *Nrf2* deficiency synergizes the deleterious effect of *miR-144/451* gene deletion on mouse erythropoiesis, we crossed *miR-144/451* KO mice with *Nrf2* KO animals. Unexpectedly, no obvious differences in the majority of anemic indices in *Nrf2/miR-144/451* double-mutant mice were observed when compared to *miR-144/451* single-KO mice (Table 1). The severity of splenomegaly (Figure 1C,D) and the accumulation of nucleated erythroid cells in spleen (Figure 1E,F) were not greater in *Nrf2/miR-144/451* double-KO mice than in *miR-144/451*-deficient mice. These results indicate that *Nrf2* deficiency does not coordinate *miR-144/451* gene deletion to exacerbate anemia during stable erythropoiesis in mice. 

Both Nrf2 and *miR-144/451* prevent the accumulation of ROS in erythroid cells [4,16]. To measure the ROS levels in erythrocytes, we stained erythrocytes with the redox-sensitive dye DCFH-DA and examined the ROS accumulation using a flow cytometer. We observed a significant increase in the ROS levels in peripheral RBCs in *Nrf2* single-KO, *miR-144/451* single-KO and *Nrf2/miR-144/451* double-KO mice compared with WT controls (Figure 1G,H). However, a marked increase in ROS levels was detected in erythrocytes in double-KO mice compared with *Nrf2* or *miR-144/451* single-deficient mice (Figure 1G,H). These results indicate that lacking either *Nrf2* or *miR-144/451* elevates ROS in erythrocytes. These results also demonstrate that *Nrf2* and *miR-144/451* prevent ROS accumulation by different and complementary mechanisms.

### 3.2. miR-144/451 and Nrf2 KO Mice Differentially Respond to PHZ-Induced Acute Hemolytic Anemia

**Figure 2:**
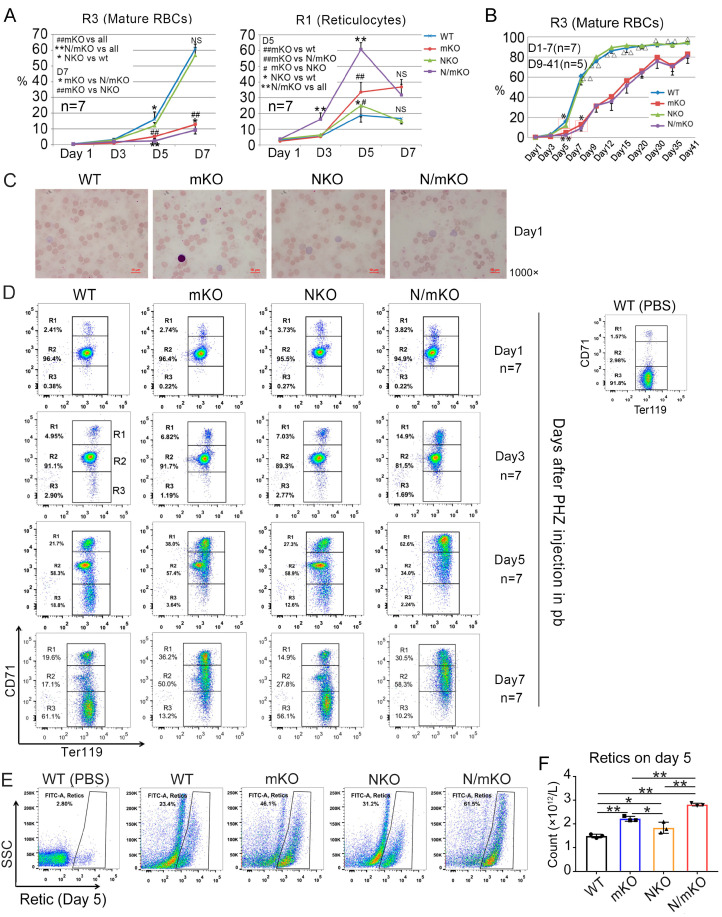
Phenotype change during recovery from acute hemolytic anemia after in vivo phenohydrazine (PHZ) injection. (**A**) Flow cytometric analysis of the mature RBCs and newly produced reticulocytes in peripheral blood 1–7 days after PHZ injection. n = 7 mice of each genotype. * *p* < 0.05, ** *p* < 0.01; # *p* < 0.05, ## *p* < 0.01 (*t*-test). (**B**) Time-course study using flow cytometry to show the percentages of mature RBCs (Ter119^+^CD71^−^) after PHZ induction for 41 days. (**C**) Peripheral blood cells stained with Wright-Giemsa on day 1. (**D**) Flow cytometry showing the mature RBCs (Ter119^+^CD71^−^) and early reticulocytes (Ter119^+^CD71^high^) in peripheral blood 1–7 days after PHZ administration. (**E**) Flow cytometry analysis of Retic-Count-Reagent-stained peripheral blood cells on day 5 after PHZ administration. PHZ-unstained WT peripheral blood cells as normal cell control. (**F**) Reticulocyte count. Note: reticulocyte count = reticulocyte percentage × total RBC number. n = 3. * *p* < 0.05, ** *p* < 0.01.

PHZ is a strong oxidant and in vivo injection of PHZ induces the rapid lysis of mature RBCs and thus acute hemolytic anemia [17,18]. To compare the phenotypic status of mice harboring inactivated *miR-144/451* or *Nrf2* we treated age-matched *Nrf2* and *miR-144/451* KO mice with a single dose of PHZ. Twenty-four hours later, almost all erythrocytes were destroyed in all genotype mice, leaving only a few intact erythrocytes in the circulating blood (day 1 in Figure 2A,C and top panel of Figure 2D). The production of new erythrocytes started quickly, evidenced by a portion of Ter119^+^/CD71^high^ cells shown in blood on day one after PHZ injection. Notably, slightly more reticulocytes appeared in *Nrf2* KO blood at this time point (Figure 2D, top panel). However, on day 5 after PHZ injection, we saw significantly more early stage reticulocytes (Ter119^+^/CD71^high^) and fewer mature RBCs (Ter119^+^/CD71^−^) in *miR-144/451* KO blood than in *Nrf2* KO blood (Figure 2A,D and Supplementary Figure S1A–C). We found similar results using Retic staining (Figure 2E,F). On day 7 after PHZ treatment, we observed a dramatic increase in mature RBCs in peripheral blood from *Nrf2* KO mice, reaching similar levels as in WT blood (Figure 2A,D). At the same time, the amount of mature RBCs in *miR-144/451* KO blood was much less than in *Nrf2* KO and WT mice, with both early (Ter119^+^/CD71^high^) and later (Ter119^+^/CD71^low^) stages of reticulocytes still very high in *miR-144/451* KO blood (Figure 2A,D). All these findings indicate that the generation of RBCs in *miR-144/451* KO but not *Nrf2* KO mice is significantly delayed during oxidant-induced erythropoiesis.

**Figure 3:**
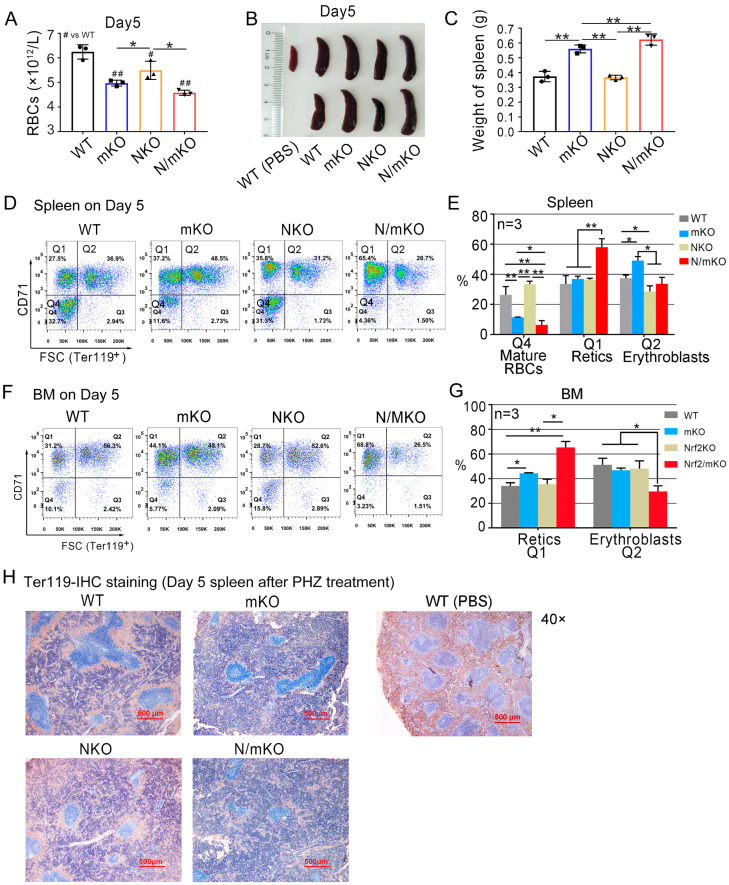
Phenotypic change in mice 5 days after PHZ administration. (**A**) Automated hematology analysis of RBCs on day 5 after PHZ injection. n = 3. # *p* < 0.05, ## *p* < 0.01; * *p* < 0.05 (*t*-test). (**B**) Spleens on day 5 after PHZ administration. All mice were 9 weeks old. (**C**) Quantitative analysis of spleen weight. n = 3. ** *p* < 0.01. (**D**) Flow cytometry gating the Ter119-positive splenic cells into three populations based on CD71 and FSC analysis on day 5 after PHZ administration. Q2: erythroblasts; Q1: reticulocytes; Q4: mature RBCs. (**E**) Quantitative analysis of the erythroblasts (Q2), reticulocytes (Q1) and mature RBCs (Q4) in panel D. n = 3. * *p* < 0.05, ** *p* < 0.01. (**F**) Flow cytometry showing the erythroid cells (Ter119 positive) in bone marrows (BM). (**G**) Quantitative analysis of the retics and erythroblasts from panel (**F**). n = 3. * *p* < 0.05, ** *p* < 0.01. (**H**) Low magnification view (40×) of immunohistochemical staining for cell surface marker Ter119. The area with brown color represents the erythroid cells. Note: much less brown area was shown in *miR-144/451* single-KO and *Nrf2/miR-144/451* double-KO spleens.

Besides reticulocytes, *miR-144/451* KO mice had less RBCs and Hb in peripheral blood (Table 2 and Figure 3A), larger spleens (Figure 3B,C) and more erythroblasts in spleens (Figure 3D,E) than *Nrf2* KO mice on day 5, though the percentages of nucleated erythroid precursors in BM were largely unaffected as shown by the flow cytometer (Figure 3F,G). Immunohistochemical analysis of Ter119-stained spleen tissues revealed that more erythroblasts and much fewer enucleated erythrocytes were in *miR-144/451* KO mice than in *Nrf2* KO mice (Figure 3H and Supplementary Figure S2). H&E staining showed a similar result (Supplementary Figure S3).

### 3.3. Inactivation of Nrf2 Provokes a Stronger Reticulocytosis in miR-144/451 KO Mice in Response to PHZ-Induced Acute Hemolytic Anemia

PHZ stimulation results in a greater defect of erythropoiesis in *miR-144/451* KO mice than in *Nrf2* KO mice (Table 2 and Figure 2); therefore, we evaluated whether *miR-144/451* and *Nrf2* synergize during PHZ-induced stress erythropoiesis. The time-course study revealed that the percentages of Ter119^+^/CD71^high^ reticulocytes (early reticulocytes) in peripheral blood were similar across all of the genotypes 24 h (1 day) after PHZ injection (Figure 2D, top panel). However, increased numbers of reticulocytes (approximately 2-fold relative to *miR-144/451* and *Nrf2* single-KO mice) in *Nrf2/miR-144/451* double-KO blood were observed on day 3 (Figure 2A,D), and on day 5 the percentage rose to approximately 60% in *Nrf2/miR-144/451* double-KO blood compared to 34% in *miR-144/451* KO and 25% in *Nrf2* KO blood (right panel of Figure 2A,D). Accordingly, the numbers of mature RBCs (Ter119^+^/CD71^−^) were much lower in *Nrf2/miR-144/451* double-KO blood than in blood from all other genotype mice (left panel of Figure 2A,D and Supplementary Figure S1A,C). These observations support that PHZ induced a more severe hemolytic anemia in *Nrf2/miR-144/451* double-KO animals.

To confirm these results, we estimated reticulocytes using flow cytometric analysis of Retic-dye-stained blood cells. Consistently, many more reticulocytes in the peripheral blood of *Nrf2/miR-144/451* double-KO mice were detected compared to *miR-144/451* KO or *Nrf2* KO mice 5 days after PHZ induction (Figure 2E,F). Moreover, larger spleens and more splenic and BM reticulocytes were found in *Nrf2/miR-144/451* double-KO mice compared to *miR-144/451* or *Nrf2* single-KO mice (Figure 3B–G).

Interestingly, the massive reticulocytosis in *Nrf2/miR-144/451* double-KO mice quickly faded and reached the levels in *miR-144/451* single-KO mice on day 7 (Figure 2A,D). Afterward, the reticulocytosis and especially the production of mature RBCs in *Nrf2/miR-144/451* double-KO mice exhibited a very similar pattern to *miR-144/451* single-KO mice, while *Nrf2* KO mice followed a pattern similar to WT mice (Supplementary Figure S4). Of note, the stress erythropoiesis in *Nrf2/miR-144/451* double-KO and *miR-144/451* single-KO mice was far worse than that in *Nrf2* single-KO and WT mice, indicating a stronger effect of *miR-144/451* deficiency than *Nrf2* inactivation on oxidant-induced murine erythropoiesis.

### 3.4. More ROS Accumulation in Newly Generated Erythrocytes from Nrf2/miR-144/451 Double-KO Mice upon PHZ Induction

**Figure 4:**
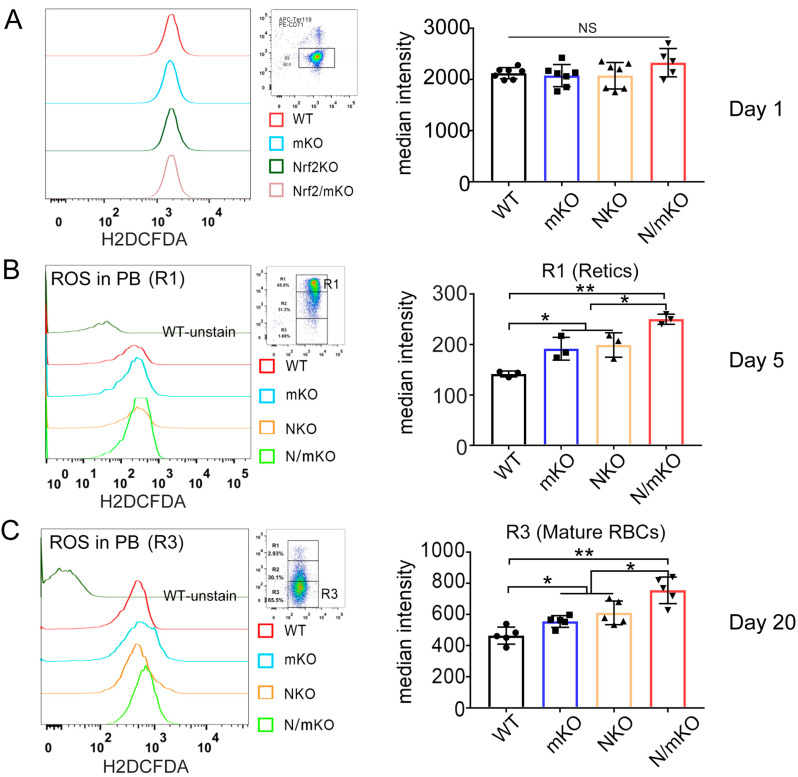
Detection of ROS in erythrocytes from peripheral blood during recovery from acute hemolytic anemia. (**A**) Flow cytometry showing ROS levels in ghost-like cells on day 1 after PHZ treatment. Right panel showing the quantitative analysis of ROS levels based on the median intensity of the DCFDA fluorescence on flow cytometer. (**B**) ROS levels in newly generated erythrocytes in region 1 (R1). Cells were from day 5 after PHZ injection. (**C**) ROS levels in mature erythrocytes in region 3 (R3). Cells were from day 20 after PHZ treatment. Note: loss of *Nrf2* and *miR-144/451* had a significant additive effect on the ROS accumulation in newly produced erythrocytes. * *p* < 0.05, ** *p* < 0.01.

We observed a dramatic accumulation of ROS in damaged RBCs in all four groups of mice 24 h after PHZ treatment (Figure 4A). Contrary to the clear accumulation patterns, the ROS levels in peripheral erythrocytes from double-KO and *miR-144/451* or *Nrf2* single-KO mice varied during the early recovery stage of acute anemia, presumably due to the heterogeneity of erythrocyte population. However, the ROS levels in newly generated double-KO erythrocytes were significantly higher than in *miR-144/451* or *Nrf2* single-KO RBCs on day 5 and 20 after PHZ injection (Figure 4B,C), indicating an additive effect of *miR-144/451* and *Nrf2* deficiency on ROS accumulation in erythrocytes during PHZ-induced acute anemia.

### 3.5. Altered Expression of miR-144/451-Targeted and Nrf2-Transcribed Detoxifying Genes

**Figure 5:**
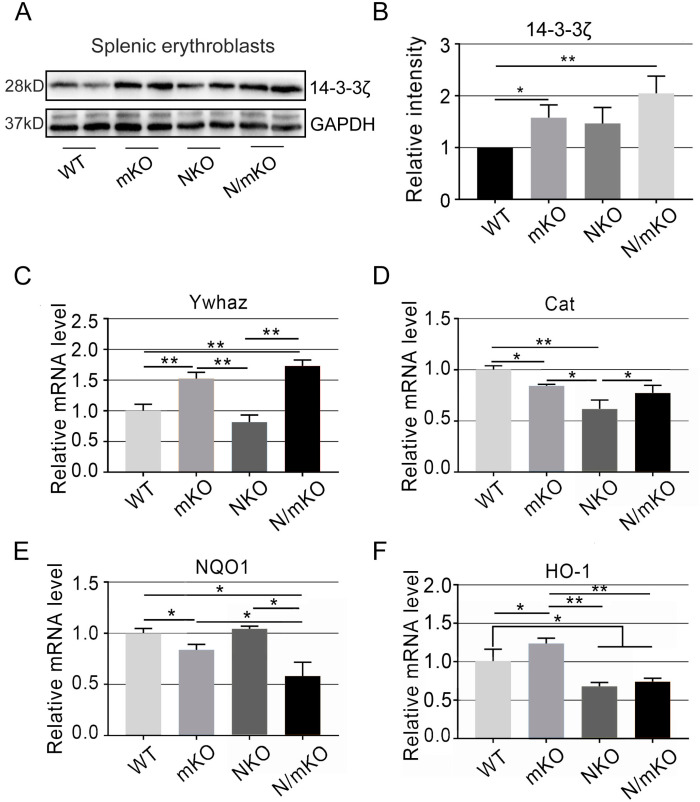
Expression levels of *miR-144/451* and Nrf2 targets in splenic erythroblasts 5 days after PHZ induction. (**A**) Western blot showing the protein levels of 14-3-3z, a direct target gene of *miR-451* in nucleated erythroid cells from spleens on day 5 after PHZ injection. (**B**) Quantitative analysis of protein levels shown in panel (**A**) using ImageJ software. qRT-PCR showing the relative mRNA levels of *Ywhaz* (**C**), *Cat* (**D**), *NQO1* (**E**), and *HO-1* (**F**) in nucleated erythroid cells from spleens on day 5 after PHZ injection. * *p* < 0.05, ** *p* < 0.01.

Catalase (*Cat*) is a transcribed gene of the antioxidant protein Foxo3 [19], a transcription factor required for detoxifying ROS during erythropoiesis [20]. We have previously reported that Foxo3 is sequestered in the cytoplasm by 14-3-3z, an adaptor protein directly inhibited by *miR-451* [4]. A deficiency of *miR-144/451* in erythroid cells leads to the derepression of 14-3-3z and enhances the sequestration of Foxo3 and thus the downregulation of Cat expression [4]. Nrf2 binds to antioxidant response elements (AREs) in its target genes and positively controls the expression of genes encoding antioxidant enzymes including NAD(P)H quinone dehydrogenase 1 (*NQO1*) and Heme Oxygenase-1 (*HO-1*) [21,22]. To evaluate the potential crosstalk between *miR-144/451* and Nrf2, we examined the expression of the genes targeted by *miR-144/451* and Nrf2 in splenic erythroblasts isolated on day 5 after PHZ stimulation. In agreement, 14-3-3z was upregulated in *miR-144/451*-deficient erythroblasts 5 days after PHZ injection (Figure 5A–C). Double-mutant erythroid cells also expressed higher 14-3-3z (Figure 5A–C). As expected, *Cat* mRNA decreased in all mutant genotypes (Figure 5D). Interestingly, *NQO1* was not downregulated in *Nrf2* KO but in double-KO erythroid cells (Figure 5E). In contrast, *HO-1* decreased in both double-KO and *Nrf2* single-KO erythroid cells (Figure 5F). All these findings suggest that the targeting mechanisms by which *miR-144/451* and Nrf2 regulate PHZ-induced erythropoiesis are not overlaid.

## 4. Discussion

In this study, we find that deleting part of the *miR-144/451* gene locus in mice makes the recovery period of PHZ-induced hemolytic anemia much longer than the abolishment of Nrf2 activity, indicating that *miR-144/451* is more important than Nrf2 for the recovery from oxidant-induced anemia. *miRNA* is a class of small non-coding RNAs mediating post-transcriptional gene regulation via Watson–Crick base pairing [23]. *miR-451* and *miR-144*, like all other *miRNAs*, are predicted to directly downregulate hundreds of genes (Targetscan.org), among which *Ywhaz, Cab39* and *Myc* have been verified in our laboratory [11,15,24]. Nrf2 also directly controls the expression of hundreds or even thousands of genes either positively or negatively, which is a typical feature shared by all transcription factors. While the effects of *miRNAs* on gene regulation are often mild, usually by coordinated tuning of a gene network, transcription factors often function as primary switches to generate profound biological effects [25]. Although Nrf2 has been identified as a crucial factor in mitigating the anemia caused by high ROS in erythroid cells [5,6], our finding that targeted deletion of the *miR-144/451* gene locus makes PHZ-induced acute anemia more severe than the inactivation of Nrf2 activity in mice strongly suggests that *miRNAs* could also be major driving forces for disease development.

Our investigation based on in vivo mouse models provides insights into the crosstalk between two antioxidant systems, *miR-144/451* and Nrf2, in maintaining redox homeostasis in erythroid cells. However, the underlying mechanism of why the depletion of *miR-144/451* makes PHZ-induced hemolytic anemia more severe than the disruption of Nrf2 remains to be elucidated. One possible mechanism is that *miR-144/451* constrains the translocation of transcription factor Foxo3 from the cytoplasm into the nucleus [4]. It has been reported that GATA1 transcriptionally activates both Foxo3 [26] and *miR-144/451* [8]. Moreover, Foxo3 amplifies GATA1-mediated transcription of genes encoding components of the autophagy machinery [26], and GATA1/Foxo3 cooperates to repress expression of Exosc8, a pivotal component of the exosome complex [27], both of which are necessary for erythroid differentiation. Such an intertwined network puts *miR-144/451* as one of the “central hubs” whose disruption is responsible for more defects from stress erythropoiesis than Nrf2 inactivation. The alternative mechanism might be that Nrf2 not only scavenges ROS but also promotes the proliferation of erythroblasts (unpublished data). In this case, loss of Nrf2 is beneficial for the differentiation of erythroid progenitors.

We, as well as other researchers, have previously demonstrated that both *miR-144/451* and Nrf2 play vital roles in reducing ROS levels and thus mitigating the oxidant damage of erythroid cells [1,4,6]. *miR-144/451* directly represses 14-3-3z, the Foxo3-binding partner, and allows the translocation of Foxo3 from the cytoplasm to the nucleus to transcribe antioxidant enzyme gene *Cat* and *Gpx1* in erythroid cells [4]. Nrf2 protects oxidant-induced erythroid cell death by directly upregulating the expression of antioxidant factors NQO1 and HO-1 in mice [22]. Strikingly, the experiments in this study identify only subtle additive effects of the targeted deletion or inactivation of *miR-144/451* and *Nrf2* genes on the severity of hemolytic anemia induced by PHZ. Moreover, this minor synergy of *miR-144/451* and *Nrf2* deficiency appears only in a very short recovery period, and Nrf2 is indeed largely dispensable during the majority of the recovery process of PHZ-induced acute anemia. One possible explanation for the lack of apparent coordination between *miR-144/451* and *Nrf2* during stress erythropoiesis is that *miR-144/451* and Nrf2 control two distinct antioxidant systems (*miR-144/451*/Foxo3/Cat/Gpx1 vs. Nrf2/NQO1/HO-1) with certain functional overlay. It is also possible that *miR-144/451*- rather than Nrf2-mediated gene regulation provides the dominant protection of erythroid cells due to other homeostatic functions of *miR-144/451*, such as sustaining erythroid differentiation and viability, as we previously reported [11,15].

PHZ is an oxidant reagent for generating acute hemolytic anemia with characteristics of damaged RBCs in severe β-thalassemia [28,29]. PHZ usually causes acute anemia, but few researchers have carried out detailed time-course studies to analyze the kinetic facilitation of the genes with phenotypic changes during oxidant-induced erythropoiesis. In the present study, we show that an additive phenotypic change, especially reticulocytosis, in *Nrf2*/*miR-144/451* double-KO mice occurred during days 3 to 7 after PHZ injection. These findings indicate that a prolonged observation upon oxidant stimulation warrants the understanding of dynamic gene regulation during specific developmental stages or the disease development of erythroid cells.

In summary, we find that the loss of both non-coding *miR-144/451* and transcription factor *Nrf2*, two genes of ROS-scavenging ability, synergizes to boost ROS levels in erythroid cells without additive anemic consequence at baseline. However, loss of *miR-144/451* and *Nrf2* genes additively affects stress erythropoiesis induced by oxidant PHZ during days 3–7 of the recovery period. In addition, the defect of erythropoiesis induced by PHZ lasts longer in *Nrf2*/*miR-144/451* double-KO and *miR-144/451* single-KO mice than in *Nrf2* KO mice. Our study confirms the crosstalk between *miR-144/451*- and Nrf2-controlled antioxidant networks during stress erythropoiesis; however, the outcome of the interaction is developmental-stage-dependent. We should keep in mind that our study relied solely on mouse models, which may not fully represent the complexity of human erythropoiesis and anemia, although Nrf2, mature *miR-144* and *miR-451* are highly conserved across mice and humans. All these issues highlight the need for further research to fully understand the underlying mechanisms and potential therapeutic implications.

## Figures and Tables

**Table 1:** CBC at baseline.

Index	WT	mKO	NKO	N/mKO
RBC (×10^12^/L)	9.58 ± 0.06	8.51 ± 0.89 *	9.53 ± 0.19	8.64 ± 0.74 *
Hb (g/L)	137.5 ± 3.87	124.25 ± 1.71 *	132.7 ± 12.09	124.33 ± 2.52 *
HCT (%)	46.43 ± 1.2	42.28 ± 1.73 **	44.07 ± 4.8	45.65 ± 0.69 #
RDW (%)	17.87 ± 0.06	23.15 ± 1.42 **	19.15 ± 0.85 *	23.18 ± 0.17 **
MCV (fl)	50.6 ± 0.88	45.46 ± 2.31 **	50.57 ± 1.42 ##	47.45 ± 0.49 **
MCH (pg)	14.75 ± 0.34	13.45 ± 0.87 *	15.1 ± 0.48 ##	13.75 ± 0.21 *
MCHC (g/L)	293.75 ± 4.57	295.75 ± 6.6	294.75 ± 3.3	290.67 ± 2.08
n	4	5	5	4

(RBC) Erythrocyte count; (Hb) hemoglobin concentration; (HCT) hematocrit; (RDW) red cell distribution width; (MCV) mean corpuscular volume; (MCH) mean corpuscular hemoglobin; (MCHC) mean corpuscular hemoglobin concentration. Experiment was repeated 3 times. * vs. WT, # vs. mKO, * *p* < 0.05, ** *p* < 0.01, # *p* < 0.05, ## *p* < 0.01.

**Table 2:** CBC on day 5 after PHZ injection.

Index	WT	mKO	NKO	N/mKO
RBC (×10^12^/L)	6.25 ± 0.29	4.98 ± 0.12 **	5.5 ± 0.37 *#	4.63 ± 0.04 **
Hb (g/L)	140.67 ± 4.93	109.33 ± 2.31 **	133.5 ± 3.54 #	102.5 ± 3.54 **#
HCT (%)	41.43 ± 0.67	40.33 ± 1.53	38.1 ± 0.28 *	39.5 ± 0.71 *
RDW (%)	37.67 ± 2.08	37.33 ± 2.08	37.85 ± 1.2	31 ± 1.41 *#
MCV (fl)	67 ± 4.59	81.3 ± 5.2 *	62.87 ± 3.53 ##	84.95 ± 1.91 **
MCH (pg)	24.13 ± 0.25	22 ± 0.61 **	23.33 ± 1.04	21.75 ± 0.35 **
MCHC (g/L)	361.67 ± 22.01	271 ± 13.08 **	372 ± 38.43 ##	255 ± 1.41 **
n	3	3	3	3

* vs. WT, # vs. mKO, * *p* < 0.05, ** *p* < 0.01, # *p* < 0.05, ## *p* < 0.01.

## Data Availability

Data are available upon request.

## Supplementary Materials

The following supporting information can be downloaded at https://www.mdpi.com/article/10.3390/genes14051011/s1, Table S1: Primers for qRT-PCR; Figure S1: Reticulocytosis at day 5 after PHZ induction; Figure S2: Immunohistochemical staining for cell surface marker Ter119 in spleen tissues on day 5 after PHZ induction; Figure S3: H&E staining of spleen tissues on day 5 after PHZ administration; Figure S4: Analysis of reticulocytosis in 4 genotypes of mice after PHZ injection.
