# Supplementary material for: Crosstalk between miR-144/451 and Nrf2 during Recovery from Acute Hemolytic Anemia
Source: Genes (Basel). 2023 Apr 29;14(5):1011. doi: 10.3390/genes14051011 (PMC10218191; doi:10.3390/genes14051011)
Supplement: Supplementary file 1 [file genes-14-01011-s001.zip › genes-2255893-supplementary.pdf]

Crosstalk between *miR-144/451* and Nrf2 during Recovery from Acute Hemolytic Anemia

Supplementary Data

Supplementary Table S1. Primers for qRT-PCR.

qRT-PCR	Primer (5'-3')	Product Size
Cat	F-CTGAAGGATCCTGACATGGT	135 bp
	R-GAAGGTGTGTGATCCATAGC	
NQO1	F-AAGAAGCTGGAAGCTGCAGA	138 bp
	R-GTTGTCGTACATGGCAGCAT	
HO-1	F-ACAGAGGAACACAAAGACCAG	136 bp
	R-GTGTCTGGGATGAGCTAGTG	
Ywhaz	F-CTGCAACGATGTACTGTCTC	160 bp
	R-CTGTGACTGGTCCACAATTC	
Gapdh	F-AATGGTGAAGGTCGGTGTGA	232 bp
	R-CTCCTGGAAGATGGTGATGG	

Supplementary Figures

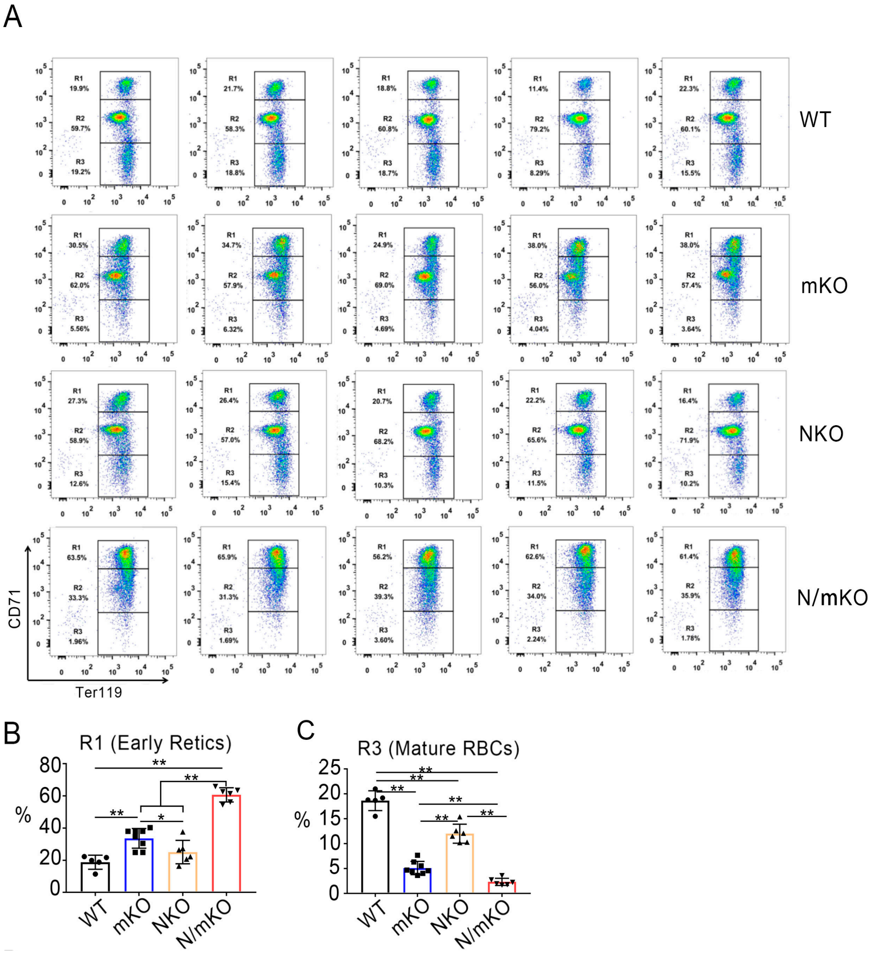

Supplementary Figure S1. Reticulocytosis at day 5 after PHZ induction. (**A**) Flow

cytometric analysis of $Ter119^{+}CD71^{+}$ reticulocytes in peripheral blood. (**B**) Quantitative analysis of the early reticulocytes in region R1 from panel A. n=5. (**C**) Mature RBCs gated in region 3. **P*<0.05, ***P*<0.01.

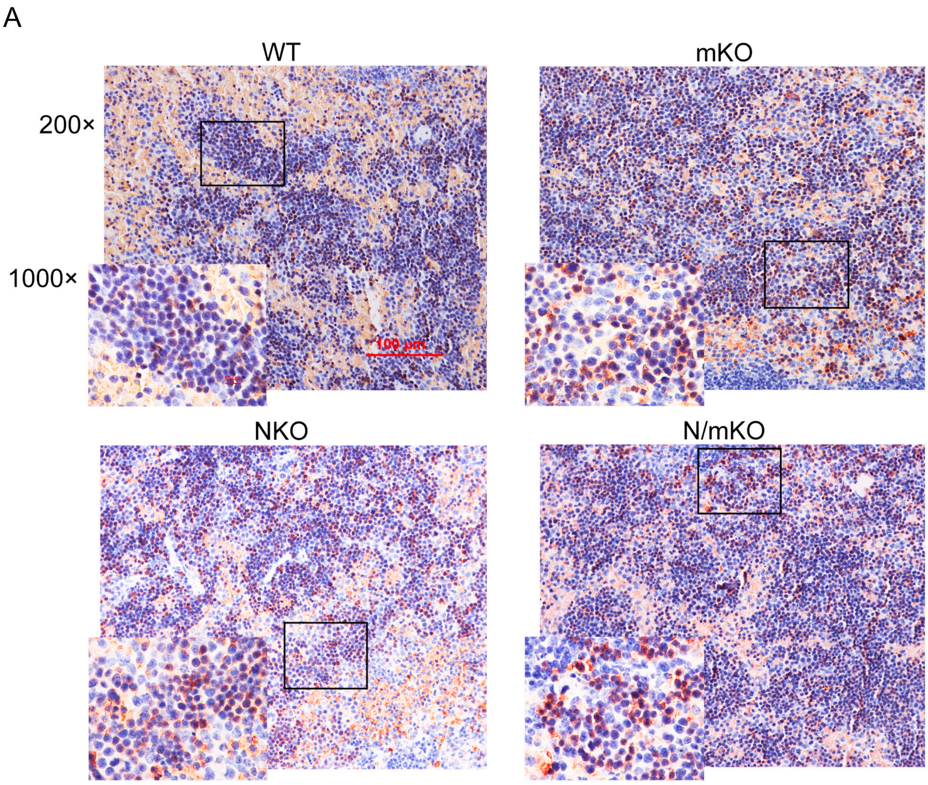

Supplementary Figure S2. Immunohistochemical staining for cell surface marker Ter119 in spleen tissues on day 5 after PHZ induction. High magnification view (200x). Insert (1000x) represents tissues in squares. The area with brown color represents the erythroid cells. Note: much less brown area was shown in *miR-144/451* single-KO and *Nrf2/miR-144/451* double-KO spleens.

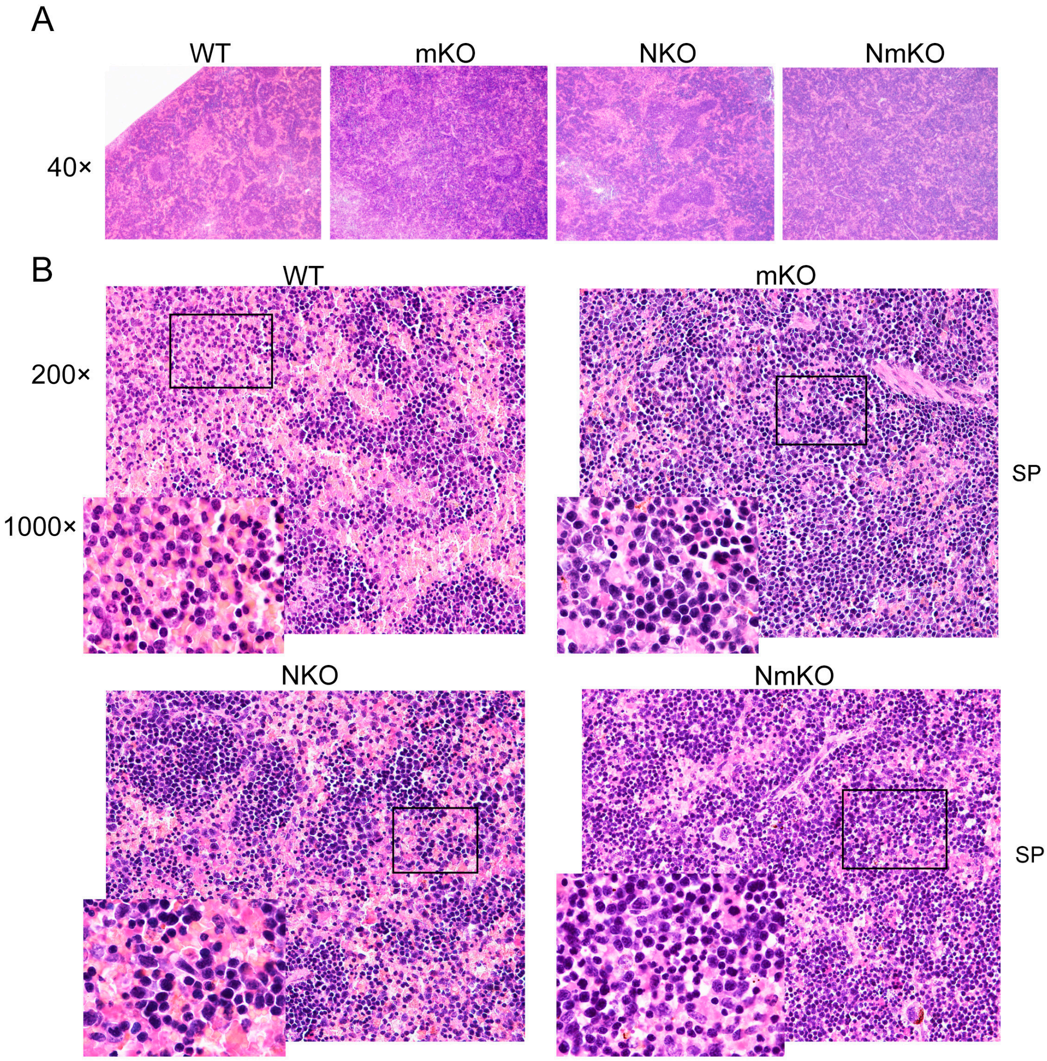

Supplementary Figure S3. H&E staining of spleen tissues on day 5 after PHZ administration. (**A**) Low magnification (40x) of H&E-stained section of spleen tissues. (**B**) High magnification images (200x). Inserts (1000x) represent the tissues in squares.

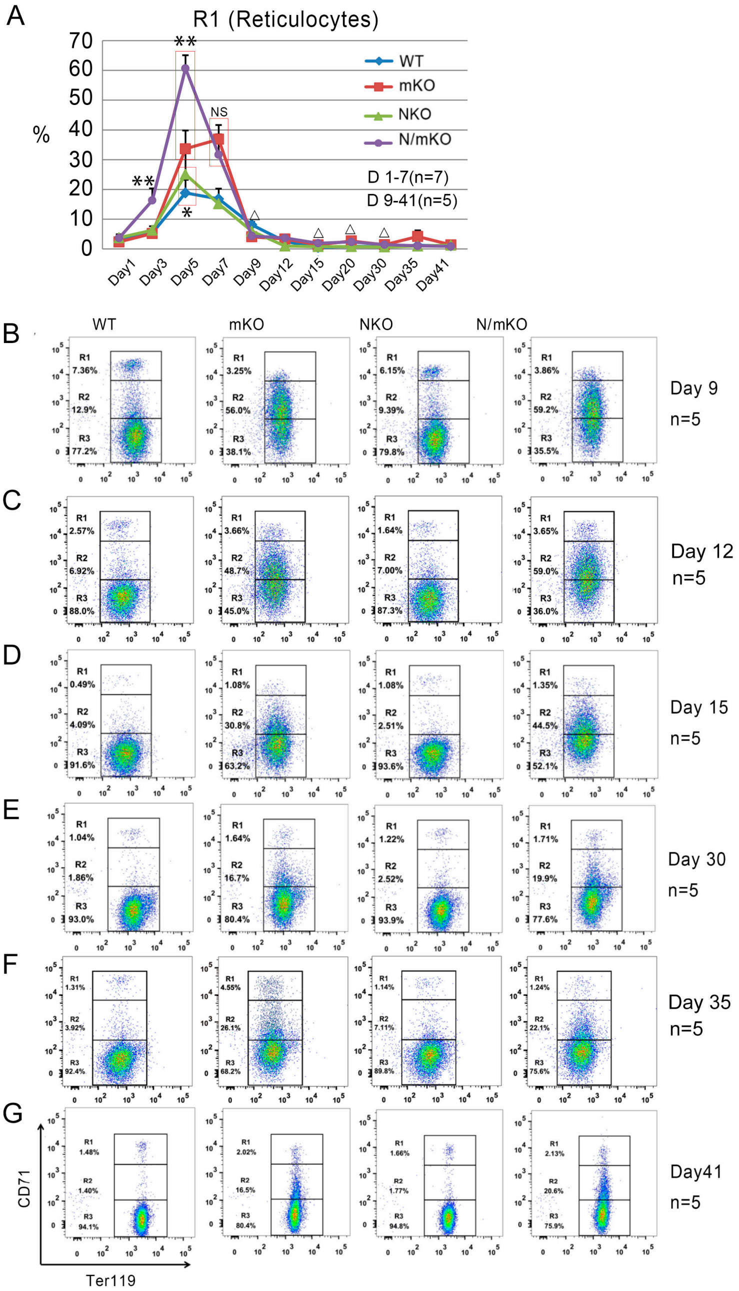

Supplementary Figure S4. Analysis of reticulocytosis in 4 genotypes of mice after PHZ injection. (**A**) Time-course study using flow cytometry to show the percentages of early reticulocytes (Ter119^{+}CD71^{high}) after PHZ induction for 41 days. (**B**–**G**) Flow cytometric analyses of the reticulocytes and their maturation during PHZ-induced stress erythropoiesis. Note: both *miR-144/451* single-KO mice and *Nrf2/miR-144/451* double-KO mice committed delayed maturation of erythrocytes.
